# Protruding Structures on Caterpillars Are Controlled by Ectopic *Wnt1* Expression

**DOI:** 10.1371/journal.pone.0121736

**Published:** 2015-03-27

**Authors:** Mina Edayoshi, Junichi Yamaguchi, Haruhiko Fujiwara

**Affiliations:** Department of Integrated Biosciences, Graduate School of Frontier Sciences, University of Tokyo Bioscience, Kashiwa, Chiba, Japan; USDA-ARS, UNITED STATES

## Abstract

Spine-like or protruding structures, which may be aposematic for predators, are often observed in multiple segments of lepidopteran larvae (caterpillars). For example, the larvae of the Chinese wheel butterfly, *Byasa alcinous*, display many protrusions on their backs as a warning that they are toxic. Although these protrusions are formed by an integument lined with single-layered epidermal cells, the molecular mechanisms underlying their formation have remained unclear. In this study, we focused on a spontaneous mutant of the silkworm, *Bombyx mori*, *Knobbed*, which shows similar protrusions to *B*. *alcinous* and demonstrates that *Wnt1* plays a crucial role in the formation of protrusion structures. Using both transgene expression and RNAi-based knockdown approaches, we showed that *Wnt1* designates the position where epidermal cells excessively proliferate, leading to the generation of knobbed structures. Furthermore, in the *B*. *alcinous* larvae, *Wnt1* was also specifically expressed in association with the protrusions. Our results suggest that *Wnt1* plays a role in the formation of protrusions on the larval body, and is conserved broadly among diverse species in Lepidoptera.

## Introduction

**Fig 1:**
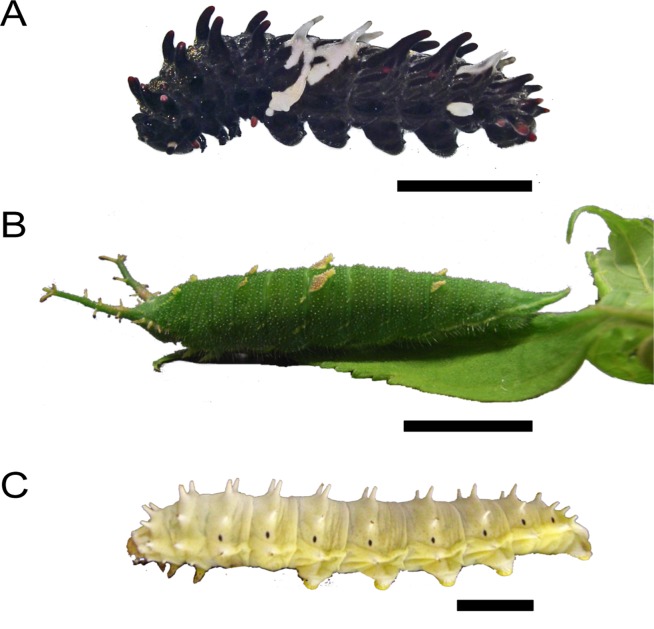
Various lepidopteran larvae with epidermal protrusion. (A) *Byasa alcinous*, (B) *Hestina assimilis* and (C) *Samia cynthia pryeri*. Scale bars, (A to C) 10 mm.

Insects have evolved various ways to avoid predation. For example, some lepidopteran species exhibit aposematic colors and shapes on their larval bodies, such as pairs of protruding structures in *Byasa alcinous* (Fig. 1A) and spot pigmentation in some *Papilio* species [1, 2], which may involve their defensive strategies. Protruding structures on the larval body are among the conserved structures characterized in troidine swallowtails (Lepidoptera: Papilionidae) and are often also observed in distantly related species such as the nymphalid butterfly, *Hestina assimilis* (Fig. 1B), and ailanthus silkworm *Samia cynthia pryeri* (Fig. 1C). Insect integument consists of a cuticle produced from a single-layered epidermis [3], and the functional and evolutionary benefits of altering its colors and morphological features are well documented [4–8]. However, how the protrusions have been gained or lost during evolution is largely unknown.

**Fig 2:**
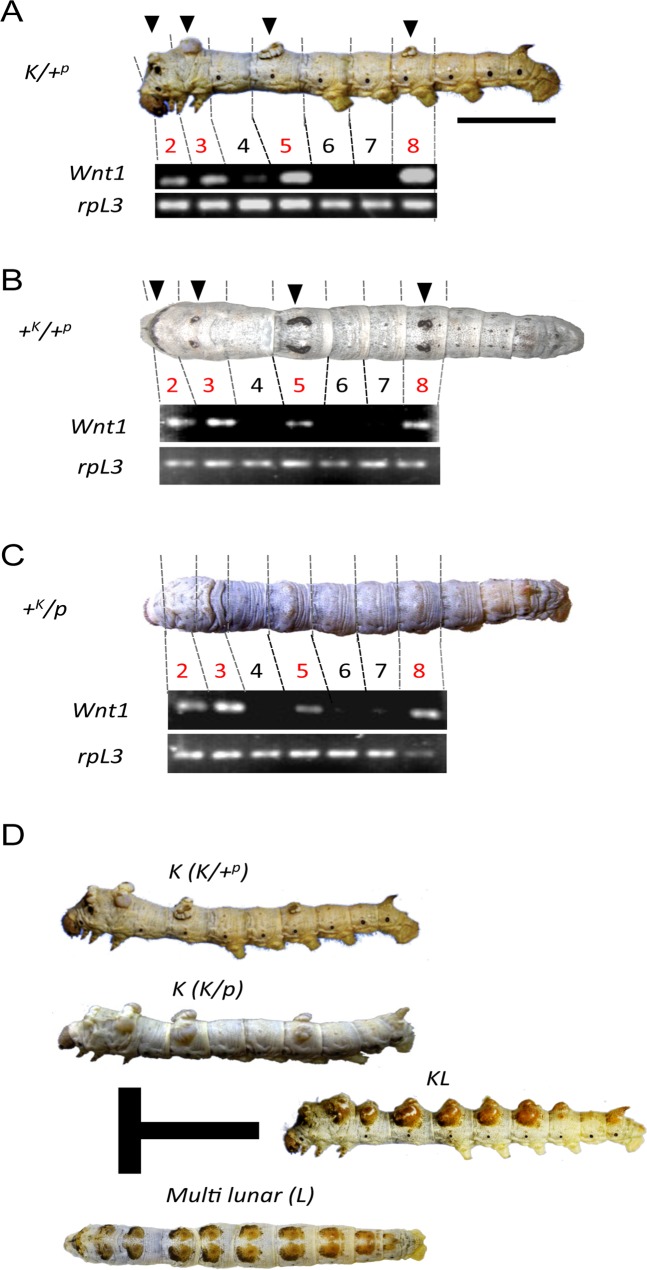
The mutant phenotype of *B*. *mori* and *Wnt1* expression in each segment. Photos of the respective *B*. *mori* mutant and WT strains at the 5^th^ instar stage. *Wnt1* expression (RT-PCR) in each segment of a 4^th^ instar *Knobbed* (*K*) (A), wild-type (WT; *+* ^*p*^) (B), and *p* strains (*p*) (C) larva. Arrowheads indicate segments with twin knobs in *K* and twin spots in WT. *rpL3*, internal control. (D) *K* strains (*K/+* ^*p*^ and *K/p*) have protrusions (knobs) in specific segments and *multi-lunar (L)* has spot markings in almost all segments. Knobs appear at all spot markings of *L* in the F1 hybrid between *K* and *L* (*KL*). Scale bar, (A) 10 mm.

In the silkworm, *Bombyx mori*, hundreds of spontaneous mutants have been obtained, many of which involve larval characteristics. *Knobbed* (*K*), one of these larval mutants, shows characteristic knobs (protrusions) paired on specific dorsal regions in the 2^nd^, 3^rd^, 5^th^ and 8^th^ body segments (Fig. 2A). The *K* phenotype is dominantly inherited and has been mapped as a single locus at 25.4 cM on the 11^th^ linkage group [9], http://www.shigen.nig.ac.jp/silkwormbase/ViewAllLinkageMap.do). Previous studies have indicated that the knobbed structures are formed by excessive proliferation of the epidermal cells [10–12], but the underlying molecular mechanism for the abnormal cell proliferation has not yet been established.

There is a question of how the pairs of knobbed structures in the *K* strain are controlled to appear in the specific dorsal regions of the 2^nd^, 3^rd^, 5^th^ and 8^th^ segments. To answer this question, larval pigmentation patterns in the silkworm provide a potential clue. It is known that the silkworm *p* locus comprises multiple alleles such as *p* (*plain*) and +^*p*^ (*normal pattern*, wild-type), that governs larval markings at the specific segments [13]. Wild-type (WT: +^*p*^) larvae have twin spot markings on the dorsal side of the 2^nd^, 3^rd^, 5^th^ and 8^th^ segments (Fig. 2B), while *p* larvae have no visible markings (Fig. 2C). The positions of the larval markings in the +^*p*^ larvae coincide with those of knobbed structure in the *K* strain. Furthermore, crossing the +^*p*^ strain with the *K* strain yields an F1generation with knobbed structures that localize with the larval markings [14] (Fig. 2A-C). However, the knobbed structures are also observed in the 2^nd^, 3^rd^, 5^th^, and 8^th^ segments in F1 larvae after crossing a *p* strain (no pigmentation) with the *K* strain (http://www.shigen.nig.ac.jp/silkwormbase/ViewStrainDetail.do?id=272). This indicates that knobbed structure formation is associated with the dorsal positions in the larval segments rather than *p* locus dependent pigmentation.

In addition, when *K* is crossed with the mutant *multi lunar (L)*, which has twin spots in consecutive dorsal segments, knobbed structures are observed in all spot markings of the F1 hybrid [14] (Fig. 2D). We have recently found that the positions of the spot markings in *L* are determined by the ectopic expression of *Wnt1* in the epidermis [15]. These observations motivated us to investigate the relationship between larval protrusion formation and *Wnt1* expression in both the silkworm *B*. *mori* and a distantly related lepidopteran insect, *B*. *alcinous*. We describe here the possibility that *Wnt1* plays an important role in the formation of protruding structures on the larval body, providing insights into a class of molecular mechanisms affecting ecologically important larval characteristics conserved among Lepidoptera.

## Materials and Methods

### Experimental insects

*B*. *mori* strains n51 (*K* phenotype), g01 (*L* phenotype) and f39 (*+* ^*K*^ */+* ^*L*^) were obtained from Kyushu University (http://www.shigen.nig.ac.jp/silkwormbase/index.jsp). F1 larvae with the *K* phenotype were obtained by crossing n51 (*K*) × f39 (*+* ^*K*^), n51 (*K*) × p^Sm^872 (*+* ^*K*^) or n51 (*K*) × N4 (*+* ^*K*^). Strains p^Sm^872 and N4 were provided by Dr. Osamu Ninaki (Tokyo University of Agriculture and Technology) and Dr. Toru Shimada (University of Tokyo), respectively. *B*. *alcinous* were collected in the Hongo Campus of the University of Tokyo. No permits were necessary to collect *B*. *alcinous*. The field collections for this project did not involve endangered or protected species.

### Transgene expression

Ectopic *Wnt1* expression using electroporation-mediated and piggyBac-based somatic transgenesis was performed as described in previous reports [15, 16]. The expression vector for *Wnt1* was the same as previously described [15]. Vector (0.5–1.0 μl) at a concentration of 2 μg μl^−1^ was injected into the haemolymph of 2^nd^ instar larvae with glass needles using a microinjector with helper plasmid PHA3PIG (methods are shown in S1 Fig. in detail). Soon after the injection (within about 3 minutes), electrical stimulation was applied to the larva using two spatially-separated droplets of phosphate-buffered saline (PBS) as electrodes (five square pulses of 20–25 V, 280 ms width).

### RNA interference

A short interfering RNA (siRNA) for *Wnt1* targeting the sequence 5′-AAGAAGATTGGCCAGAGAGAA- 3′ was designed using siDirect version 2.0 (http://sidirect2.rnai.jp) according to the criterion [17, 18] (purchased from FASMAC Co., Japan). For negative controls, the Universal Negative Control siRNA (Nippon Gene Co., Japan) was used. siRNAs (0.5 μl; 250 μM) were injected into the hemolymph of 3^rd^ instar larvae with glass needles using a microinjector. To introduce siRNA into the region only around one side of the knob [16], soon after the injection, PBS droplets were placed near the injection site and knobbed region in the 5^th^ larval segment and electrical stimuli applied as described above (S1 Fig.).

### Quantitative and semi-quantitative RT-PCR

cDNAs were synthesized from the larval epidermis of *B*. *mori* in the 4^th^ larval stage and *B*. *alcinous* in the 4^th^ and 5^th^ larval stages. After anaesthetization of the larvae on ice, the epidermis was dissected in cold PBS. When dissecting the epidermis, only the dorsal part was used, subcutaneous tissues such as muscle and fat body were trimmed away carefully with forceps. Total RNA was extracted using TRI reagent (Sigma) and purified with standard phenol/chloroform extraction following treatment with DNase I (TaKaRa) for 15 min at 37°C. The RNA was quantified by spectrophotometry (NanoDrop, Thermo Scientific) and the *A*_260_/*A*_280_ ratio confirmed to be 1.8–2.0. Total RNA (0.1–1 μg) was used in the reverse transcriptase reactions, which were performed per the manufacturer’s instructions using random hexamers and M-MuLV reverse transcriptase (GE Healthcare; First-Strand cDNA Synthesis Kit).

Quantitative RT-PCR was performed using the StepOne system with the Power SYBR Green PCR master mix (Applied Biosystems) using a MicroAmp Fast Optical 48-well reaction plate (Wako, Japan) under the manufacture’s recommended condition (denaturation at 95°C for 10 min, followed by 40 cycles of 95°C for 15 s, 60°C for 60 s). Relative gene expression values were estimated using relative standard curve method. The primers for *B*. *alcinous* for *Wnt1* were qBa_wnt1_01 (5′-GATTCCGATTCAGCCGGGAGTTCGT-3′, 5′-ACATGCGCTCTGCCGGCTTCGTT-3′) and qBa_rpL3_01 (5′-GACCGTATGGGCAGAACATATGTCTG-3′, 5′-TCTTGCTTGACTTAG TAAAGGCCTTCTTC-3′) for ribosomal protein L3 (*rpL3)* used as an internal control [7, 8, 19] (S1 Table). All primers were designed to target products < 120 bp using Primer Express 2.0 software (Applied Biosystems). Standard curves were generated for all primer pairs to estimate efficiency and to confirm the limit of detection. The specificity of primer pairs was confirmed by melting curves.

In *B. mori*, semi-quantitative RT-PCR was performed using the following primers: cWnt1_02-F (5′-GGCGGTTCACGCTACGCTA-3′) and cWnt1_05-R (5′-ATCCACAATTTTTCCGAACAAGTT-3′) for *Wnt1*, and rpL3-5 (5′-AGCACCCCGTCATGGGTCTA-3′) and rpL3-3 (5′-TGCGTCCAAGCTCATCCTGC-3′) for an *rpL3* internal control [13, 15]. The amplification program was as follows: 95°C for 2 min, then 35 cycles of 95°C for 15 s, 55°C for 30 s, 72°C for 30 s and final extension 72°C for 4 min.

## Results

### Knobs in the silkworm *K* mutant correlate with *Wnt1* expression

We first tried to clarify by semi-quantitative RT-PCR the expression of *Wnt1* in the epidermal segments of +^*p*^ and *p* larvae. We found that *Wnt1* was expressed in the 2^nd^, 3^rd^, 5^th^ and 8^th^ segments if both +^*p*^ larvae, which have twin spot markings, and *p* larvae which lack pigmentation (Fig. 2B, C). We further examined *Wnt1* expression in a *K* strain with a +^*p*^ allele background (*K*/+^*p*^ strain) and found that *Wnt1* expression coincided with the knob structures in the 2^nd^, 3^rd^, 5^th^ and 8^th^ segments which had pairs of knobs (Fig. 2A). Although weak expression of *Wnt1* was observed in the 4^th^ segment, which does not have the knob structure, we speculate that the limited *Wnt1* expression was insufficient for protrusion formation. Since the *K* strain with a *p* allele background (*K*/*p*) also forms knobs in the four segments (Fig. 2D), we think that *Wnt1* expression in those specific segments is correlated with the knob formation rather than pigmentation. Recently, we reported that *Wnt1* expression was observed in all segments with spot markings in the *L* strain. The fact that the F1 hybrid of *L* and *K* (*K*/*L*) has knobbed structures in all spot markings (Fig. 2D) supports this. Since it was shown that a *cis*-regulatory change of *Wnt1* expression is responsible for the *L* phenotype [15], we hypothesized that region-specific *Wnt1* expression in the epidermis caused the protruding integument in the *K* strain.

### Ectopic *Wnt1* expression induces knobs in the *K* mutant

**Fig 3:**
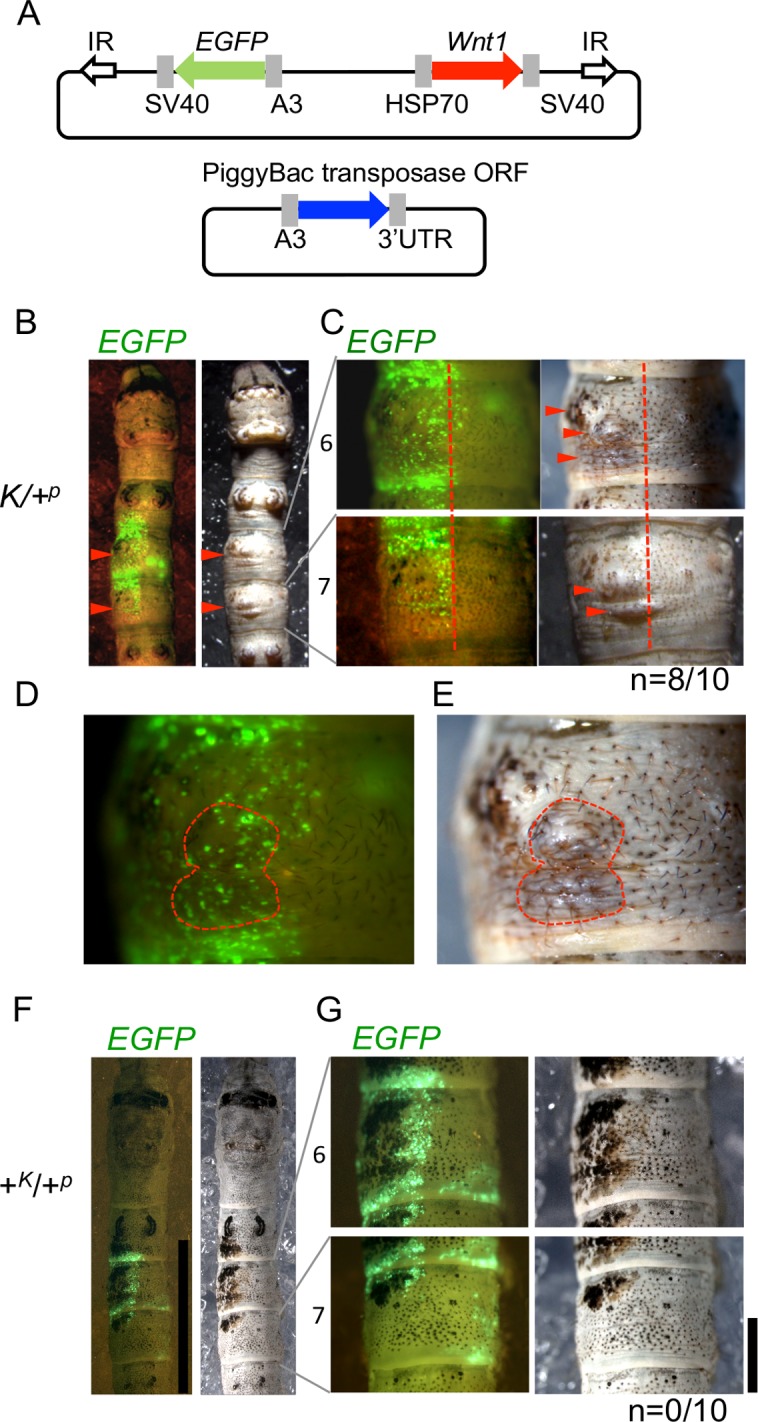
*Wnt1* in epidermis generates protruding structure. (A) Schematic of the vectors used in the *Wnt1* transgene experiment. The *EGFP-Wnt1* expression vector is shown above; the piggyBac transposase helper plasmid is shown below. Gray boxes show promoters, terminators and 3′UTR. IR represents the piggyBac recognition sequence. (B) Transgene expression of *EGFP* and *Wnt1* by electroporation in *Knobbed* (*K*) at the 5^th^ larval stage. (C) Enlargement of the 6^th^ and 7^th^ segments in (B). Ectopic *Wnt1* expression induced additional knobs (red arrowheads) around corresponding regions where *EGFP* positive cells were observed. (D, E) Further enlargement of the 6^th^ segment in (C). The ectopic knob is indicated by dotted red line. (F) Transgenic expression of *Wnt1* in the wild-type (WT) strain. A 5^th^ instar larva of the WT (*+* ^*p*^) strain, which ectopically expressed *Wnt1* and *EGFP* in the same manner as described above. (G) Enlargement of the 6^th^ and 7^th^ segments. Note that no knobs appeared in spite of evident *Wnt1* expression which is confirmed by the brown and black pigmentation around the *EGFP* positive cells. The number of individuals on which knobbed structures appeared among all individuals that survived to the 5^th^ instar is shown at the bottom of the panel in (C) and (G). Scale bars, (F) 10 mm; (G) 2 mm.

To test this hypothesis, we used a novel technique, electroporation-mediated and piggyBac-based somatic transgenesis [16], which enables genes of interest to be stably expressed in arbitrary regions where the plasmid has been incorporated in local tissue sites (S1 Fig.). We injected a plasmid containing both *Wnt1* and *EGFP* as a visible marker along with a piggyBac helper plasmid into the hemolymph of 2^nd^ instar larvae and applied electroporation to drive the plasmids into individual cells [16] (Fig. 3A). Following ectopic expression of *Wnt1* within the left side of *K* larval segments typically devoid of structures (i.e. 6th and 7th segments), knobbed structures co-localized with regions of the epidermis generating *EGFP* signals in 80% of the tested individuals (Fig. 3B, C, red arrowheads and S2 Table). The knobs and epidermal pigmentation generated by ectopic *Wnt1* expression also were apparent in regions distinct from major *EGFP* signals (Fig. 3C). Since electroporation also introduces plasmid DNA into cells that comprise subcutaneous tissues such as fat body and muscle, the strong *EGFP* signals around the protruded and pigmented regions (e.g. right side signals) were likely derived from non-epidermal cell transformation. *Wnt1* expression in these subcutaneous tissues should not contribute to protrusion nor pigmentation formation in the epidermis. Enlargement of the protrusion/pigmentation regions in the *K*/+^*p*^ strain (Fig. 3D, E, inner area within dotted red line), revealed multiple *EGFP* signals that likely were derived from epidermal cells. In similar ectopic experiments examining spot making formation in the *L* mutant, we recently showed that the *Wnt1* protein is excreted from the *EGFP* positive cells and functions as a morphogen to cause pigmentation around the cells [15]. We thus speculate that the protrusion and pigmentation observed in this study is similarly derived from the few epidermal cells positive for *EGFP* expression. The resulting ectopic knobs as well as the ordinary ones enlarged until the final larval stage and then shrank in the pupal and adult epidermis. This suggested that ectopically expressed *Wnt1* induces the development of the formation of knobs in any dorsal region of the *K* epidermis. In contrast, the introduction of *Wnt1* and helper plasmids into the WT strain (+*K*/+^*p*^) failed to induce ectopic knobs, although ectopic pigmentation was clearly observed as previously reported (Fig. 3F, G and S2 Table). These results demonstrate that *Wnt1* determined the positions not only for larval spot markings in the WT (*+* ^*p*^) strain but also for knobs in the *K* mutant.

### *Wnt1* expression is essential for knobs in the *K* mutant

**Fig 4:**
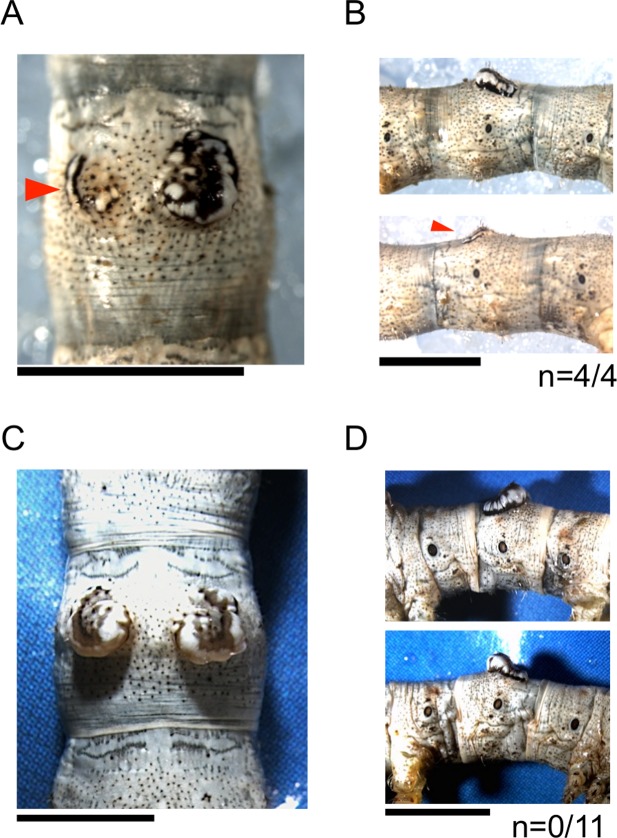
Effects of *Wnt1*-RNAi on the formation of knobs at the 5^th^ larval stage. (A, B) The left knob in the 5^th^ segment was suppressed by siRNA introduced by electroporation (red arrowheads), whereas no effects were observed in the right knob (A and the upper panel in B). (C, D) Introduction of negative control siRNA (Universal Negative Control SiRNA (Nippon gene)) by electroporation in the 5^th^ segment of the *K* mutant. No effects were observed on the formation of knobs. (D) Right and left lateral views of another individual in the same experiment. All knobs were also intact. The number of individuals in which knobs were repressed among all individuals that survived to the 5^th^ instar is shown at the bottom of the panel in (B) and (D). Scale bars, (A to D) 5 mm.

The requirement of *Wnt1* for the formation of knobs was further tested using RNAi. Since it is known that siRNA or dsRNA are not introduced effectively into cells merely by injection in most of lepidopteran larva, we used a novel electroporation mediated method that enables effective RNAi in the silkworm larva [16]. When *Wnt1* targeting siRNA was introduced into the knobbed region of the left side in the 5^th^ segment of the *K* strain by electroporation, we observed that the formation of knobs were suppressed only on that side in all individuals that we tested (Fig. 4A, B and S2 Table). The larval pigmentation in the left knobbed region was also repressed compared with the right side pattern. The electroporation mediated knockdown usually results in mosaic phenotype [16] and thus a fraction of the pigmentation remained in the left region (Fig. 4A). However, there were no effects on the knobbed structure or pigmentation when the Universal Negative Control siRNA was introduced into the left side of the *K* strain (Fig. 4C, D and S2 Table). These results indicated that, in the *K* mutant, *Wnt1* is both necessary and sufficient for the knob formation.

### *Wnt1* expression associated with larval protrusion in *B*. *alcinous*

**Fig 5:**
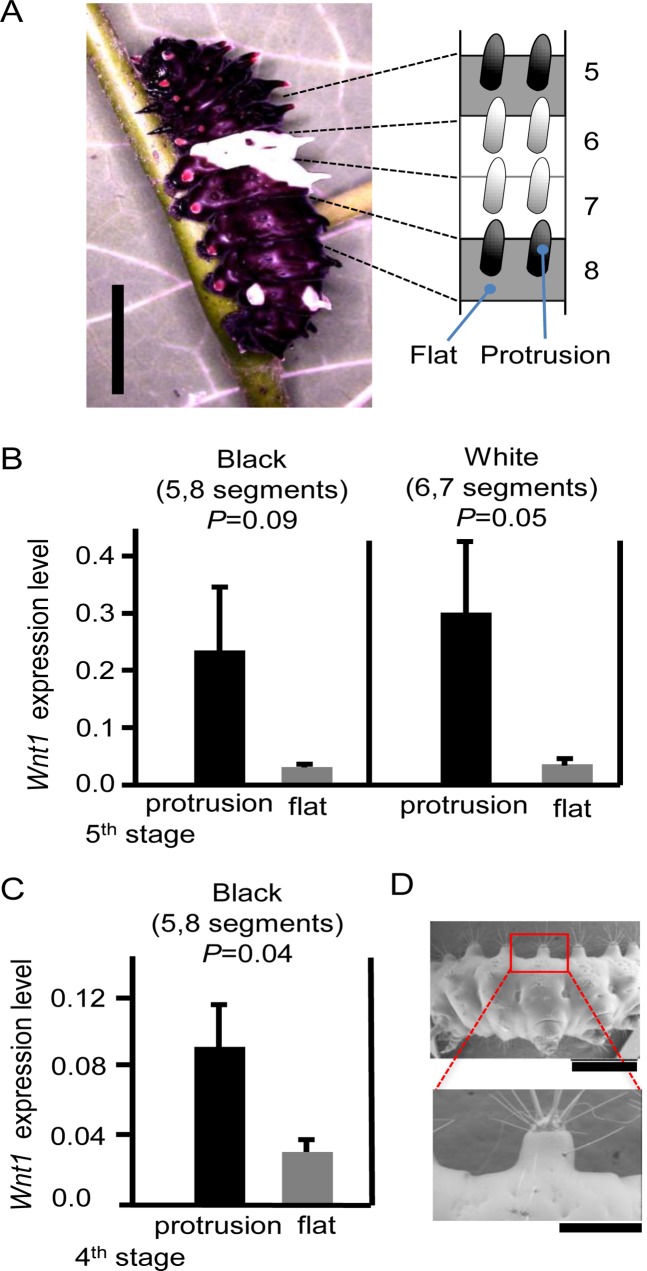
*Wnt1* expression in the epidermis of *Byasa alcinous* larvae. (A) A 5^th^ instar larva of *B*. *alcinous* and schematic of protrusions from the dorsal view. Expression levels of *Wnt1* in the protruded regions and the flat regions of the 5^th^ (B) and 4^th^ (C) instar larvae of *B*. *alcinous*. *P* values were based on paired *t*-test (one-tailed). Error bars show S.D. based on three or four biological replicates. (D) Scanning electron micrographs (SEMs) of the protruding structures in the 1^st^ instar larva of *B*. *alcinous*. Scale bars, (A) 10 mm; (D) 1 mm for top and 300 nm for bottom panels.

Next, we investigated whether epidermal *Wnt1* was associated with larval protrusions in the lepidopteran species *B*. *alcinous*. We measured *Wnt1* expression in the protruding regions and the flat regions by RT-PCR (Fig. 5A). Our results showed a tendency that *Wnt1*expression was higher in the protruding regions relative to the flat ones during the 5^th^ instar stage (Fig. 5B, *p* < 0.1). Dominant expression of *Wnt1* in the protrusion was also observed during the 4^th^ instar stage (Fig. 5C, *p* < 0.05). On the tip of protrusion in this species during the 1^st^ larval instar, we further found characteristic structures that resemble the “bulge” in the larval epidermis of *B*. *mori* (Fig. 5D). This “bulge” structure in the center of the spot markings in the *L* and +^*p*^ strains of the silkworm was reported to be associated with *Wnt1* expression [15]. These data suggested the possibility that *Wnt1* expression was also involved in forming protrusions in *B*. *alcinous*.

## Discussion

In this study, we have demonstrated that *Wnt1* designates the region where protrusions emerge on the flat cuticle of the larval body in the *K* mutant of *B*. *mori*. Some silkworm strains have paired larval markings in the 2^nd^, 3^rd^, 5^th^ and 8^th^ segments, corresponding to where *Wnt1* was expressed (Fig. 2A, B); however, *Wnt1* expression did not lead to the formation of protrusions in any silkworm strains other than *K* (Fig. 3F, G). Although we do not yet know the identity of the gene located at the *K* locus, we speculate that it may encode a factor that interacts with the *Wnt1* pathway and may subsequently lead to cell proliferation proximal to the *Wnt1* expressing region.

**Fig 6:**
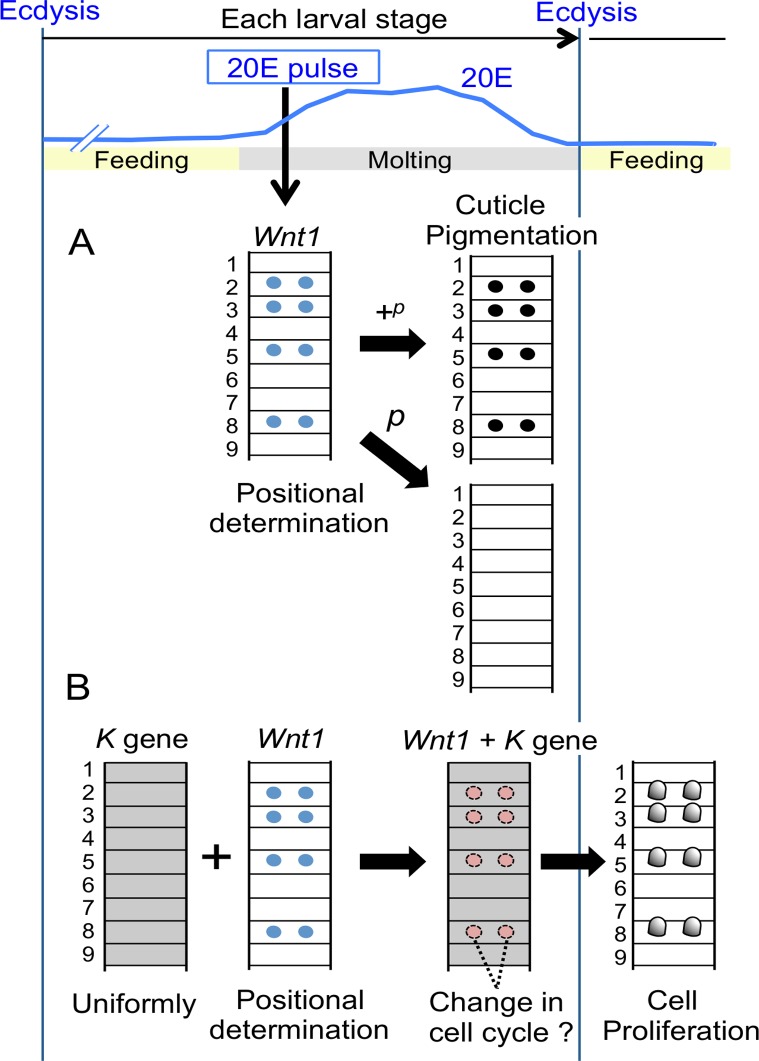
Schematic mechanism for knob formation. (A) The spatial expression pattern of *Wnt1*, which is induced by 20E pulse, determines the position of pigmentation in *B*. *mori* at each larval stage [15]. Cuticle pigmentation occurs just before ecdysis via the *+* ^*p*^ allele at the *p* locus, but with *p* [13]. (B) Knob formation is associated with *Wnt1* and 20E. According to the results of the transgene experiments in the *K* mutant, only *Wnt1* is necessary for the formation of knobs in *K*, but knobs are not formed in regions that are ectopically pigmented in the WT strain (*+* ^*K*^). This suggests that an additional factor exists in the *K* strain (*K* gene, gray), which may be the gene responsible for *K*. We hypothesize that it cooperates with *Wnt1* to form the knobs. The blue line indicates the 20E titer as described in Kiguchi *et al*. [30].

Fig. 6 summarizes the functional role of *Wnt1* in larval pigmentation and knob formation in *B*. *mori*. *Wnt1* is thought to determine the position of the twin spot markings on the 2^nd^, 3^rd^, 5^th^ and 8^th^ segments, which results in the larval pigmentation in the +^*p*^ but not in the *p* strain [13] (Fig. 6A). In the *K* strain, knob formation occurs in sites determined by *Wnt1*, regardless of +^*p*^ or *p* (Fig. 6B). Furthermore, periodic *Wnt1* expression is presumably induced by an ecdysteroid (20E) pulse during each larval instar [15]. Our experiments demonstrated that ectopic *Wnt1* expression in conjunction with the *K* gene may induce excess epidermal cell proliferation in any larval region (Fig. 3B), and we speculate that the *K* gene may be uniformly expressed in the entire dorsal epidermis (Fig. 6B). Because it has been reported that cell proliferation in the knob regions of *K* occurs mainly after a larval molt [10, 12, 20–22], we speculate that ecdysteroid-induced *Wnt1* expression in concert with the *K* gene may induce DNA syntheses in each larval stage and that actual cell proliferation occurs after the molts (Fig. 6B).

In *B*. *alcinous*, we found that *Wnt1* was expressed more dominantly in protruded regions than in flat regions during the 4^th^ and 5^th^ instar stages (Fig. 5B, C), indicating that repeated *Wnt1* expression in each larval stage may be involved in protrusion formation in this species. Similarly, it was reported that periodic *Wnt1* expression in each larval stage in response to 20E is involved in spot marking formation of the *L* strain of the silkworm [15]. In *B*. *alcinous*, white and black pigmentation are observed in whole epidermal regions across some segments (Fig. 5A), but we think that its coloration may be controlled by some pathways other than the *Wnt1* pathway, in contrast to the spot marking formation. We also observed a characteristic “bulge” structure on the tip of each protrusion in *B*. *alcinous* larvae (Fig. 5D). It has been reported that ectopic epidermal *Wnt1* expression is correlated with the “bulge” structure in spot marking formation in the silkworm [15]. In addition, the larval protrusions in the silkworm *K* strain and *B*. *alcinous* are composed of integument lined with a single-layer of epidermal cells. Based on the similar features observed in *B*. *alcionus* and *B*. *mori*, we speculate that *Wnt1* controls larval protrusion formation in both species, although further functional evidences are necessary for protrusion formation in *B*. *alcinous*. We also sought to knockdown *Wnt1* expression by *in vivo* electroporation in *B*. *alcinous*, but have not yet observed a phenotype, mainly due to the high lethality in this insect. It would be interesting to see if a similar correlation between *Wnt1* expression and the appearance of epidermal protrusions also occurs in *B*. *alcinous* and other species, such as those shown in Fig. 1.

The location of larval protrusions in *B*. *alcinous* is analogous to that of the aposematic orange spots observed in larva of the old world swallowtail, *Papilio machaon*. Recently, we also found an association between *Wnt1* expression and spot markings in this species [15]. These observations suggest a relationship between aposematic coloration and protrusions via *Wnt1* function even among distantly related lepidopteran larvae. *Wnt1* was first identified as an oncogene [23] for which aberrant expression results in an abnormal cell cycle [24–26]. The role of *Wnt1* in evolutionary traits such as insect spot markings has been reported more recently with the finding that spotted pigmentation on adult wings in *Drosophila guttifera* is generated by the co-option of *Wnt1* expression [15, 27–29]. These reports, along with our present study, suggest that *Wnt1* has multiple cellular roles and is crucial for inducing the production of complex body morphologies.

## Supporting Information

S1 Fig Procedure for *in vivo* electroporation. (PDF)

S1 Table List of primers used in real time PCR analyses. (PDF)

S2 Table Functional analysis of *Wnt1*. (PDF)

## References

[1] Sherratt TN, Beatty CD (2003) The evolution of warning signals as reliable indicators of prey defense. Am Nat 162: 377–389. 14582002 10.1086/378047

[2] Prudic KL, Oliver JC, Sperling FA (2007) The signal environment is more important than diet or chemical specialization in the evolution of warning coloration. Proc Natl Acad Sci U S A 104: 19381–19386. 18029450 10.1073/pnas.0705478104 PMC2148298

[3] Marian RG, Adam SW (1995) Molecular model systems in the Lepidoptera. New York, NY: Cambridge University Press 299–301 p.

[4] Wiklund C, Sillen-Tullberg B (1985) Why distasteful butterflies have aposematic larvae and adults, but cryptic pupae: evidence from predation experiments on the monarch and the European swallowtail. Evolution 39: 1155–1158. 28561515 10.1111/j.1558-5646.1985.tb00456.x

[5] Grant JB (2007) Ontogenetic colour change and the evolution of aposematism: a case study in panic moth caterpillars. J Anim Ecol 76: 439–447. 17439461 10.1111/j.1365-2656.2007.01216.x

[6] Futahashi R, Fujiwara H (2008) Juvenile hormone regulates butterfly larval pattern switches. Science 319: 1061 10.1126/science.1149786 18292334

[7] Futahashi R, Fujiwara H (2008) Identification of stage-specific larval camouflage associated genes in the swallowtail butterfly, *Papilio xuthus* . Dev Genes Evol 218: 491–504. 10.1007/s00427-008-0243-y 18712529

[8] Futahashi R, Shirataki H, Narita T, Mita K, Fujiwara H (2012) Comprehensive microarray-based analysis for stage-specific larval camouflage pattern-associated genes in the swallowtail butterfly, *Papilio xuthus* . BMC Biol 10: 46 10.1186/1741-7007-10-46 22651552 PMC3386895

[9] Tazima Y (1964) The genetics of the silkworm. London: Logos Press and Prentice Hall.

[10] Shimura S, Kiuchi M, Kiguchi K (2009) Epidermal mitotic activity associated with knob formation in the "Knobbed" mutant of the silkworm, *Bombyx mori*. J. Insect Biotechnol. Sericology 78: 165–171.

[11] Shimura S, Kiuchi M, Kiguchi K (2011) Characteristics of epidermal cells associated with knob formation in the knobbed mutant of the silkworm, *Bombyx mori*. Int. J. Wild Silkmoth & Silk 16: 77–87.

[12] Shimura S, Kiuchi M, Kiguchi K (2012) Epidermal mitosis associated with knob formation in the fifth instar larva of the knobbed mutant of the silkworm, *Bombyx mori*. J. Insect Biotechnol. Sericology 81.2: 69–73.

[13] Yoda S, Yamaguchi J, Mita K, Yamamoto K, Banno Y, Ando T et al (2014) The transcription factor Apontic-like controls diverse colouration pattern in caterpillars. Nat Commun 5: 4936 10.1038/ncomms5936 25233442

[14] Tanaka Y (1916) Genetic studies on the silkworm. Jour. of the College of Agr., Tohoku Imp. Univ., Sapporo, Japan 7 (3): 129–255.

[15] Yamaguchi J, Banno Y, Mita K, Yamamoto K, Ando T, Fujiwara H (2013) Periodic Wnt1 expression in response to ecdysteroid generates twin-spot markings on caterpillars. Nat Commun 4: 1857 10.1038/ncomms2778 23673642

[16] Ando T, Fujiwara H (2013) Electroporation-mediated somatic transgenesis for rapid functional analysis in insects. Development 140: 454–458. 10.1242/dev.085241 23250219

[17] Yamaguchi J, Mizoguchi T, Fujiwara H (2011) siRNAs induce efficient RNAi response in *Bombyx mori* embryos. PLoS One. 6: e25469 10.1371/journal.pone.0025469 21980469 PMC3184131

[18] Ui-Tei K, Naito Y, Takahashi F, Haraguchi T, Ohki-Hamazaki H, Juni A et al (2004) Guidelines for the selection of highly effective siRNA sequences for mammalian and chick RNA interference. Nucleic Acids Res 32: 936–948. 14769950 10.1093/nar/gkh247 PMC373388

[19] Futahashi R, Fujiwara H (2006) Expression of one isoform of GTP cyclohydrolase I coincides with the larval black markings of the swallowtail butterfly, *Papilio xuthus* . Insect Biochem Mol Biol 36:63–70. 16360951 10.1016/j.ibmb.2005.11.002

[20] Kato Y, Oba T (1977) Temporal pattern of changes in mitotic frequency in the epidermis and other larval tissues of *Bombyx mori* . J Insect Physiol 23: 1095–1098.

[21] Kato Y (1977) Mitotic frequency in the epidermis during early larval life of *Bombyx mori* . Zool. Mag 86: 250–253.

[22] Kawasaki H, Nishida S, Kanke E (2001) Fluctuation of the ploidy level in the epidermis of *Bombyx mori* during the penultimate and ultimate larval instars. Invertebr Reprod Dev 40: 109–116.

[23] Nusse R, van Ooyen A, Cox D, Fung YK, Varmus H (1984) Mode of proviral activation of a putative mammary oncogene (int-1) on mouse chromosome 15. Nature 307: 131–136. 6318122 10.1038/307131a0

[24] Giraldez AJ, Cohen SM (2003) Wingless and Notch signaling provide cell survival cues and control cell proliferation during wing development. Development 130: 6533–6543. 14660542 10.1242/dev.00904

[25] Logan CY, Nusse R (2004) The Wnt signaling pathway in development and disease. Annu Rev Cell Dev Biol 20: 781–810. 15473860 10.1146/annurev.cellbio.20.010403.113126

[26] Dichtel-Danjoy ML, Ma D, Dourlen P, Chatelain G, Napoletano F, Robin M et al (2013) *Drosophila* p53 isoforms differentially regulate apoptosis and apoptosis-induced proliferation. Cell Death Differ 20: 108–116. 10.1038/cdd.2012.100 22898807 PMC3524635

[27] Kronforst MR, Young LG, Kapan DD, McNeely C, O'Neill RJ, Gilbert LE (2006) Linkage of butterfly mate preference and wing color preference cue at the genomic location of wingless. Proc Natl Acad Sci U S A 103: 6575–6580. 16611733 10.1073/pnas.0509685103 PMC1458925

[28] Monteiro A, Glaser G, Stockslager S, Glansdorp N, Ramos D (2006) Comparative insights into questions of lepidopteran wing pattern homology. BMC Dev Biol 6: 52 17090321 10.1186/1471-213X-6-52 PMC1654149

[29] Werner T, Koshikawa S, Williams TM, Carroll SB (2010) Generation of a novel wing colour pattern by the Wingless morphogen. Nature 464: 1143–1148. 10.1038/nature08896 20376004

[30] Kiguchi K, Agui N (1981) Ecdysteroid levels and developmental events during larval moulting in the silkworm, *Bombyx mori* . J Insect Physiol 27: 805–812.

