# Supplementary material for: Protruding Structures on Caterpillars Are Controlled by Ectopic Wnt1 Expression
Source: PLoS One. 2015 Mar 27;10(3):e0121736. doi: 10.1371/journal.pone.0121736 (PMC4376876; doi:10.1371/journal.pone.0121736)
Supplement: S1 Fig — (PDF) [file pone.0121736.s001.pdf]

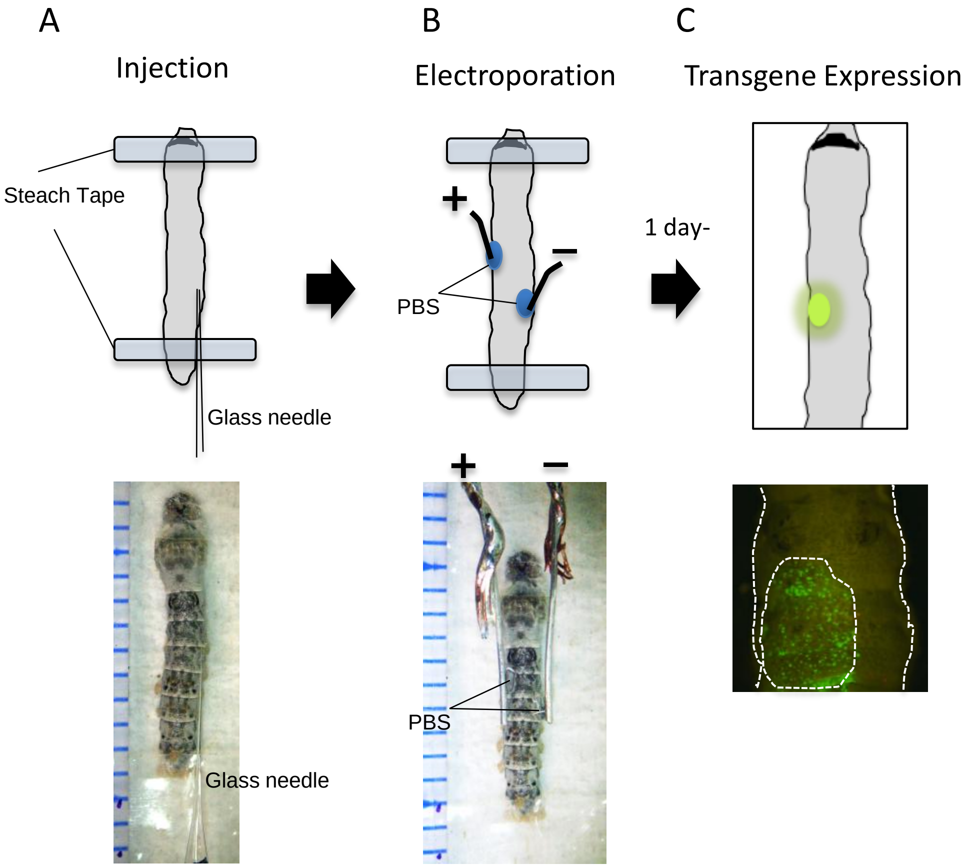

S1 Figure. Procedures of *in vivo* electroporation.

Schematics and comparative photos are shown. **(A)** Larva was Immobilized on the paper with adhesives. DNA solution (2 μg/μl) was injected into the haemolymph using a microinjector (FemtoJet; Eppendorf, Germany) and a broken-tip glass needle (GD-1; Narishige, Japan) which was prepared using a needle puller (PP-830; Narishige). The needle was inserted under the skin at a point distant from the region of interest to prevent from the damage (e.g., opposite side of the region on 7th or 8th segment). (**B**) Immediately after injection (within about 3 minutes), PBS droplets were placed between platinum electrodes and larval body, and appropriate voltage was applied. **(C)** The fluorescence of *EGFP* marker of the transgene expression was observed in the plus electrode which was largely corresponding to the region of PBS.
