# Supplementary material for: Protruding Structures on Caterpillars Are Controlled by Ectopic Wnt1 Expression
Source: PLoS One. 2015 Mar 27;10(3):e0121736. doi: 10.1371/journal.pone.0121736 (PMC4376876; doi:10.1371/journal.pone.0121736)
Supplement: S1 Table — (PDF) [file pone.0121736.s002.pdf]

S1 Table. List of primers used in real time PCR analyses.

| Target/Referernce mRNA | Name of primer | Primer sequence 5'-3' | Amplicon size (bp) | Amplification efficiency (%) |
|---|---|---|---|---|
| *Wnt1* | qBa_wnt1_01 | GATTCCGATTCAGCCGGGAGTTCGT | 100 | 101 |
| | | ACATGCGCTCTGCCGGCTTCGTT | | |
| *rpL3* | qBa_rpL3_01 | GACCGTATGGGCAGAACATATGTCTG | 98 | 97 |
| | | TCTTGCTTGACTTAGTAAAGGCCTTCTTC | | |
