# Supplementary material for: Protruding Structures on Caterpillars Are Controlled by Ectopic Wnt1 Expression
Source: PLoS One. 2015 Mar 27;10(3):e0121736. doi: 10.1371/journal.pone.0121736 (PMC4376876; doi:10.1371/journal.pone.0121736)
Supplement: S2 Table — (PDF) [file pone.0121736.s003.pdf]

S2 Table. Effects of functional analysis of *Wnt1*.

target gene	strain	lethal rate	phenotype
Transgene expression of *Wnt1*	*K*	9% (1/11)	80%* (8/10)
	WT	29% (4/14)	0%(10/10)
siRNA *Wnt1*	*K*	43% (3/7)	100%** (4/4)
	WT	27% (4/15)	0%(11/11)

*Ectopic knobs were ovserved in 80% of indivisuals that we tested.

**Knobs were repressed in all indivisuals that we tested.
